# Interdisciplinary Management of an Isolated Intrabony Defect

**DOI:** 10.1155/2014/672152

**Published:** 2014-11-23

**Authors:** Sheetal Ghivari, Anand C. Patil, Shavina Patil, Sunita Shivanand, Anukriti Tyagi

**Affiliations:** Department of Conservative Dentistry and Endodontics, KLE VK Institute of Dental Sciences, Nehru Nagar, Belgaum 590010, India

## Abstract

The treatment of intrabony defects is a real challenge in molar teeth as it is chronic, slowly progressing disease which needs timely intervention. Periodontal inflammation associated with intrabony defect is not a separate entity as it secondarily affects the pulp causing retrograde pulpitis. However, treatment of these lesions will be complicated due to extensive bone loss. The tooth was endodontically treated followed by periodontal surgery to eliminate the deep periodontal pocket and promote bone fill in osseous defect. PepGen P-15 composited with platelet rich plasma was utilized for enhancing bone formation. The combination of these graft materials provides synergistic effect on bone regeneration.

## 1. Introduction 

The periodontal therapy mainly involves scaling and root planning alone or combined with hard and soft tissue surgery. Clinicians usually face problem while treating advanced periodontal lesions whose prognosis is considerably poor [1]. Molars are the most vulnerable teeth for attachment loss and are frequently indicated for extraction. Molar teeth with three or more bony wall defects are most commonly lost if timely intervention is not done [2].

Etiology of intrabony defect involvement includes local environmental factors such as tooth position which contributes to food impaction, plaque accumulation, angular position, or position of the tooth with respect to the alveolar housing [2]. Another important factor in the etiology of the infrabony pocket is the occlusal traumatic lesion and localized juvenile periodontitis [3]. According to Goldman and Cohen, there is a direct relationship between the number of walls intact in intrabony defect and the prognosis of the therapy [4].

Today, with the advance of guided tissue/bone regeneration technology, three-wall and even two-wall defect can be treated successfully [5]. Anorganic bovine matrix when combined with synthetic cell binding peptide mimics cell binding region of type I collagen (PepGen P-15, Dentsply Friadent, Mannheim, Germany). It provides a tissue-engineered hospitable biomimetic habitat for cells like osteoblasts and fibroblasts and demonstrates an increased expression of growth factors [6].

Very few case reports are found in the literature, using combination of PepGen P-15 and platelet rich fibrin (PRF) as a graft material in successful management of intrabony defect. PRF contains multitude of growth factors which is considered to be ideal for pulp dentin complex regeneration [7]. Present case report aims to highlight successful management of tooth by endodontic therapy followed by regenerative periodontal surgical intervention using a combination of platelet rich plasma (PRF) and PepGen (P-15) as bone replacement graft.

## 2. Case Report

**Figure 1:**
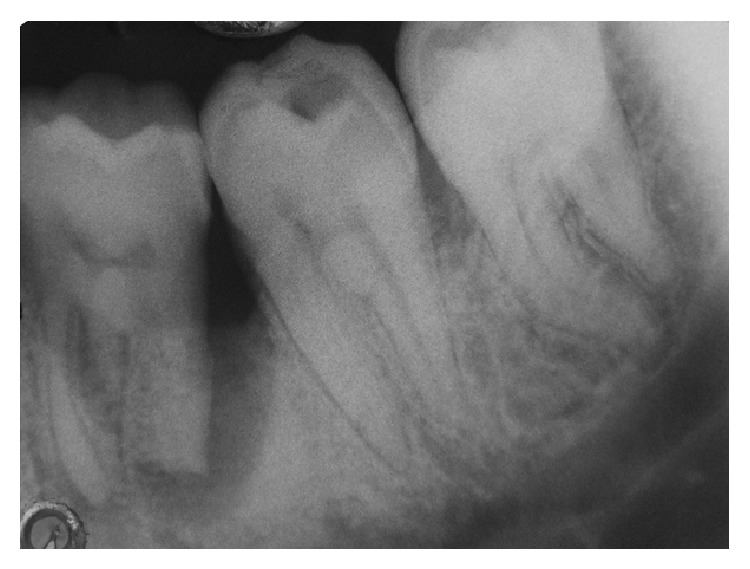
Diagnostic radiograph.

A 24 year male patient reported with the complaint of food lodgment and occasional pain in relation to right lower first molar. Clinical examination revealed deep periodontal pocket measuring 9 mm on distal aspect of 46 and no mobility (Figure 1). There were no other teeth periodontally involved in the remaining dentition. Radiographically, the tooth in question showed extensive bone loss around distal root with apical resorption (Figure 1). The bone loss in this case may be attributable to the presence of long standing periodontal pocket due to improper positioning of the teeth in the alveolar hosing. Electric pulp test and cold test revealed delayed response indicating a nonvital mandibular first molar. Based on patient's history and clinical examination, the lesion was diagnosed as combined lesion (Primary perio and secondary endo). The treatment plan involved initially endodontic therapy followed by periodontal regenerative surgery.

**Figure 2:**
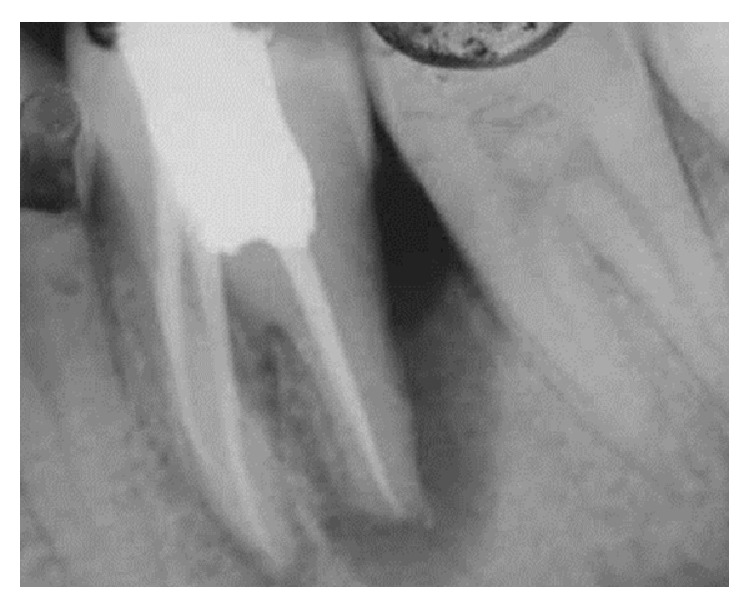
Postobturation.

**Figure 3:**
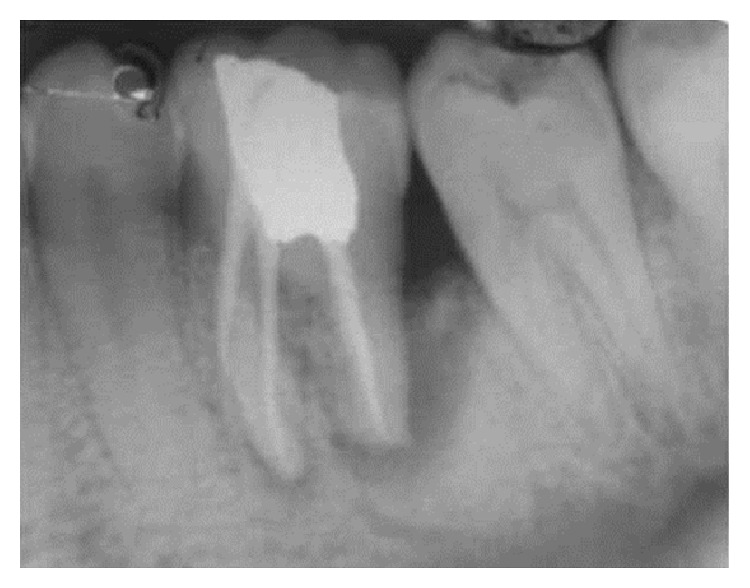
1-month follow-up.

At the initial visit an endodontic access cavity was prepared and four canals were identified followed by working length determination. Cleaning and shaping of the root canal was performed using nickel titanium rotary files with a copious irrigation of saline, 5% sodium hypochlorite solution, and 17% EDTA. Tooth was temporized after placing calcium hydroxide intracanal medication. Three weeks later, the tooth was asymptomatic. The canals were finally rinsed with normal saline and dried with absorbent points and obturation was performed using cold lateral compaction of gutta-percha using AH Plus resin sealer followed by postendodontic restoration (Figure 2). Patient was recalled after one month (Figure 3) for periodontal surgical procedure.

At the subsequent visit, the scaling and root planning was done followed by occlusal evaluation to identify any occlusal trauma which in this case was absent. Subsequently a full thickness mucoperiosteal flap was raised. Upon flap elevation, the vertical and the horizontal component of the bone loss in the defect area were measured. The vertical component of the bone loss was around 9 mm and the horizontal component was 5 mm at the roof of the furcation. The configuration of the defect was evaluated using periodontal probe (Figures 5 and 6); it was found to be three-walled bony defect (Figure 7).

25 mL of blood was drawn from the patient's antecubital vein and centrifuged for 10 min at 3000 revolutions per minute (PRF). PRF is in the form of a platelet gel and is mixed with PepGen P-15 (Dentsply Friadent, Mannheim, Germany) manipulated according to manufactures instructions and repositioned in a bony defect followed by closure of mucoperiosteal flap.

**Figure 4:**
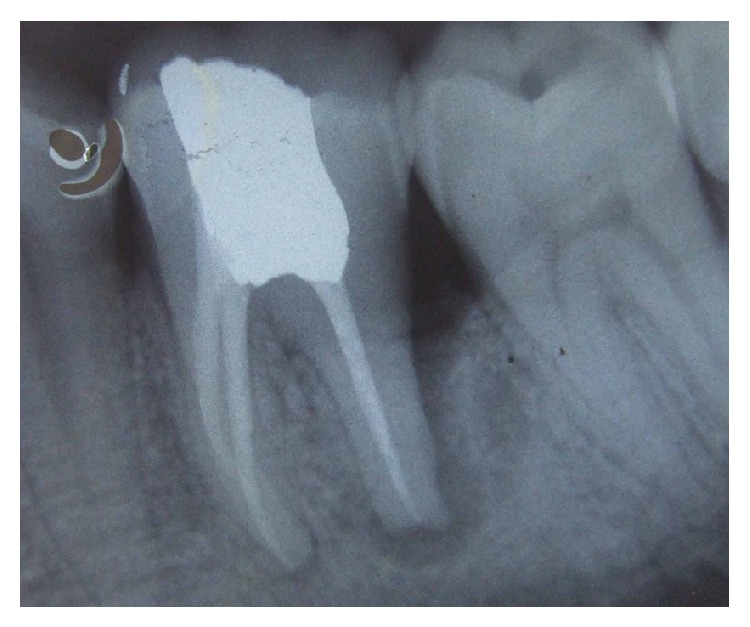
Six-month follow-up.

**Figure 5:**
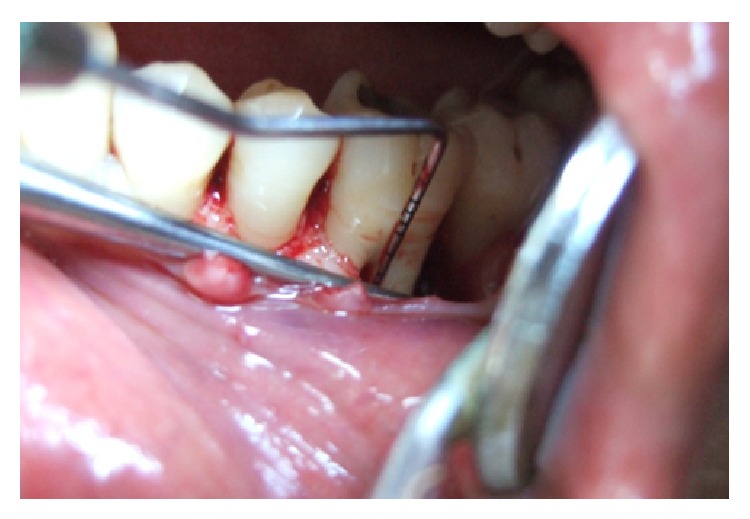
Evaluation of periodontal pocket.

**Figure 6:**
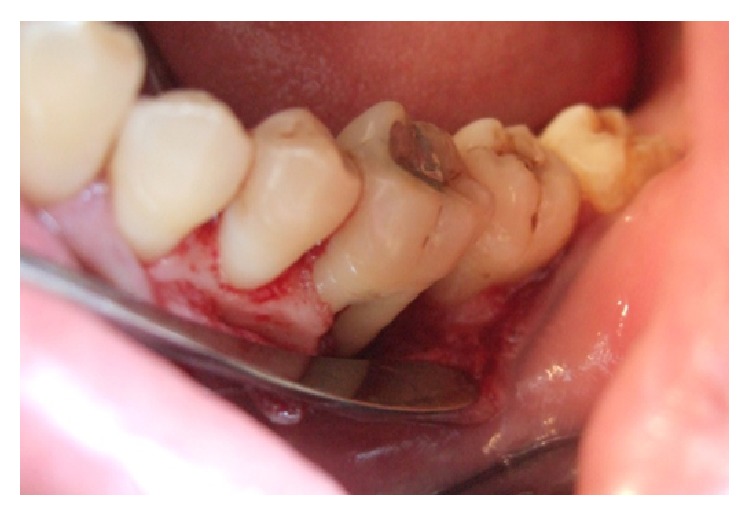
After surgical debridement.

**Figure 7:**
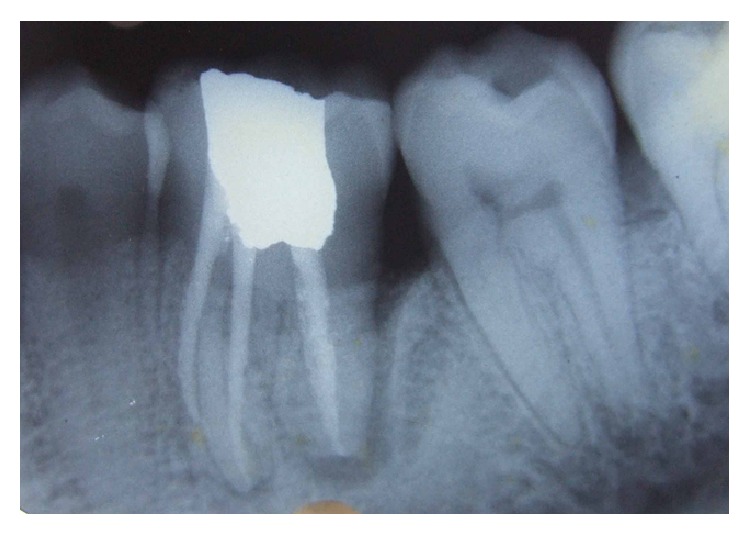
1-year follow-up.

**Figure 8:**
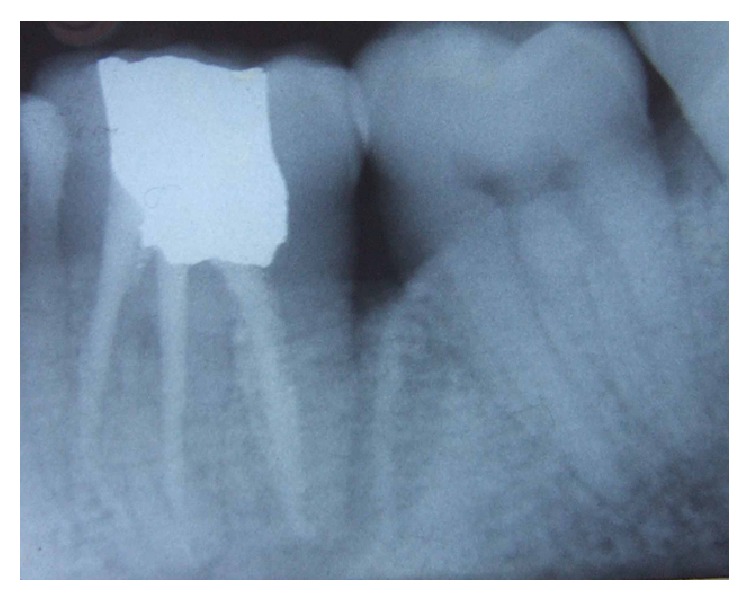
3-year follow-up.

Patient was on medication (antibiotics and analgesics) and maintenance recall schedule at three, six, and twelve months and three years. Healing was found to be satisfactory (Figures 4, 5, and 8).

## 3. Discussion

John Prichard stressed the coexistence of local environmental factors and the occlusal trauma as one of the two etiologies of the intrabony pocket [2]. The mandibular molar region is the most common site for intrabony pocket [4]. Clinical evidence indicates that intrabony defects that were treated with P-15/ABM material have shown long-term positive results with an average clinical attachment gain of 1.6 mm and 2.4 mm probing depth reduction. No adverse outcomes such as ankylosis or root resorption were noted [3, 6].

We chose Pepgen P-15 as it is osteoconductive material and acts as a scaffold on which host bone might grow. To enhance the regenerative potential, it was mixed with PRF as it is a host's own biologic tissue containing concentrated suspension of growth factors like PDGF, TGF, and IGF found in platelets. The growth factors are involved in wound healing and promoters of tissue regeneration [7, 8].

Other treatment modalities that have been used successfully are root resection and hemisection procedures. The case presented in this report was more amenable to regenerative therapy as the patient was young; more positive results of chosen therapy were anticipated. Moreover, when flap was raised, the tooth showed no mobility as there was sufficient bone support on the lingual side of the root.

Pulpal and periodontal tissues are closely related and disease transmission between these two lesions has been demonstrated by many studies [9]. The lesions of endodontic origin are treated by root canal therapy alone, if there is existing inflammatory periodontal disease than at later stage periodontal treatment is initiated [1]. In the present case the lesion is combined primarily involving the periodontium when left for long time pulp has been infected. Endodontic lesion is primarily a close environment wound which heals once the source of infection is removed. The periodontal wound is open to oral environment posing difficulty for repair and regeneration [10, 11].

Intrabony defect repair has been always a challenge to the clinician. According to Prichard analysis of wound healing, the intrabony pocket heals similar to the fractured bone repair; supplementing the wound with additional osteogenic graft materials like PepGen P-15 and PRF has provided synergetic effect on wound healing and repair [12].
